# Preconception care: preventing and treating infections

**DOI:** 10.1186/1742-4755-11-S3-S4

**Published:** 2014-09-26

**Authors:** Zohra S Lassi, Ayesha M Imam, Sohni V Dean, Zulfiqar A Bhutta

**Affiliations:** 1 Division of Women and Child Health, Aga Khan University Karachi, Pakistan

**Keywords:** preconception, infection, HIV, Sexually transmitted infections

## Abstract

**Introduction:**

Infections can impact the reproductive health of women and hence may influence pregnancy related outcomes for both the mother and the child. These infections range from sexually transmitted infections (STIs) to TORCHS infections to periodontal disease to systemic infections and may be transmitted to the fetus during pregnancy, labor, delivery or breastfeeding.

**Methods:**

A systematic review and meta-analysis of the evidence was conducted to ascertain the possible impact of preconception care for adolescents, women and couples of reproductive age on MNCH outcomes. A comprehensive strategy was used to search electronic reference libraries, and both observational and clinical controlled trials were included. Cross-referencing and a separate search strategy for each preconception risk and intervention ensured wider study capture.

**Results:**

Preconception behavioral interventions significantly declines re-infection or new STI rates by 35% (95% CI: 20-47%). Further, condom use has been shown to be the most effective way to prevent HIV infection (85% protection in prospective studies) through sexual intercourse. Intervention trials showed that preconception vaccination against tetanus averted a significant number of neonatal deaths (including those specifically due to tetanus) when compared to placebo in women receiving more than 1 dose of the vaccine (OR 0.28; 95% CI: 0.15-0.52); (OR 0.02; 95% CI: 0.00-0.28) respectively.

**Conclusion:**

Preconception counseling should be offered to women of reproductive age as soon as they test HIV-positive, and conversely women of reproductive age should be screened with their partners before pregnancy. Risk assessment, screening, and treatment for specific infections should be a component of preconception care because there is convincing evidence that treatment of these infections before pregnancy prevents neonatal infections.

## Introduction

Infections can impact the reproductive health of women and hence may influence pregnancy related outcomes for both the mother and the child. These infections range from sexually transmitted infections (STIs) to TORCH infections to periodontal disease and may be transmitted to the fetus during pregnancy, labor, delivery or breastfeeding.

STIs are a serious universal reproductive health concern with the weight of the disease falling excessively on women, especially those who are young or socioeconomically disadvantaged. The World Health Organization estimates of syphilis infection among pregnant women stands at 2 million [1]. Congenital syphilis can have devastating complications including stillbirth, premature birth, neonatal death, developmental delay, blindness, deafness and seizures. Similarly, gonorrhea during pregnancy is associated with chorioamnionitis, premature rupture of membranes, preterm labor and can potentially cause severe conjunctivitis in the newborn. Chlamydia too in the pregnant woman can have serious consequences for her neonate, including conjunctivitis and pneumonia. With timely detection and treatments prior to conception, thereby helping the mother-to-be to achieve an optimal state of health earlier, these maternal and fetal complications can be averted.

Initially, HIV/AIDS largely infected high-risk populations, such as commercial sex workers and injecting drug users. Currently, however, the demographic with the highest incidence rate is women of reproductive age [2]. Women are at particular risk of being infected in stable heterosexual relationships, since they often lack the skills to negotiate safe sexual behaviors. Approximately 15.9 million women who are HIV positive today could potentially transmit the virus to their future children [3,4]. Babies born with HIV are more likely to develop AIDS sooner and have more serious complications. Additionally, HIV-positive women are more likely to terminate their pregnancies, give birth to low birth weight (LBW) babies, deliver preterm, or experience stillbirths [5-8]. Perinatal HIV transmission still accounts for ≥90% of the cases of pediatric acquired immunodeficiency syndrome in the United States (US) [9]. Although 18-40% of women in the US become pregnant after an HIV diagnosis [10,11], forty percent of these infants are born to mothers who are unaware of their HIV status. Therefore, in addition to timely preconception screening, these women require close monitoring of the disease status and treatment protocol as the state of pregnancy does not make the disease worse and women with HIV can have healthy pregnancies. However, women with low CD4 counts or active infections may have more complications. Also, while highly active antiretroviral therapy is contraindicated in the first trimester, treatment with anti-retroviral therapy (ART) has drastically decreased the mother to child transmission in the past few decades.

Detection and treatment of STIs is inadequate without reducing risky behaviors (multiple partners, unprotected intercourse). Hence the current focus of interventions targeting STIs in women of child-bearing age, including HIV, focus on promoting safe sex behaviors and the provision of easily accessible contraception. This does not only empower women but enables them to plan their pregnancies until their infection has been eradicated (controlled in the case of HIV) and/or their treatment regimen has been optimized.

TORCH is another set of infections with serious neonatal complications, with congenital cytomegalovirus being the leading cause of hearing loss in children. The neonatal complications are more severe if acquired during early pregnancy and this necessitates early screening before the critical period of fetal organogenesis. Immunization against those infections that are vaccine-preventable would have greater benefit if they were also targeted to young women of reproductive age.

The incidence of STIs remains very high in low- and middle- income countries (LMICs) being highest in urban men and women in their second to fourth decade of life when sexual activity is highest [12]. Adolescents continue to be at high risk for acquiring an STI owing to a greater likelihood than adults of having multiple sexual partners, engaging in unprotected intercourse, selecting high-risk partners and older partners [13]. Other risky behaviour that increases the incidence of STI includes substance abuse [14]. STIs during pregnancy are associated with adverse pregnancy outcomes ranging from early abortion and premature births to congenital infections and death [15,16]. Many studies have shown between a two- and five-fold increased risk for HIV infection among persons who have other STIs [17], possibly increasing the occurrence of poor pregnancy outcomes even more.

STIs and especially HIV are a huge social stigma, compounded by lack of knowledge of safe and effective options and access to services or contraceptive products to prevent unplanned pregnancies. Options-based dialogues with their healthcare providers about integrated family planning and reproductive health care help enlighten and empower these women with regards to their reproductive choices.

This review deals with global evidence on interventions that have met with some degree of success in addressing the issue of infections in women of child-bearing age, especially STIs and HIV. It also includes current evidence on the success of vaccination, especially tetanus, in reducing neonatal deaths.

## Methods

This paper systematically reviewed all literature published up to December 2012 to identify studies describing the effectiveness of preconception (period before pregnancy and between pregnancy) interventions for prevention and management of infections such as HIV/AIDS, STIs, cytomegalovirus, and periodontal infections and their impact on maternal, newborn and child health (MNCH) outcomes. Electronic databases such as PubMed, Cochrane Libraries, EMBASE, and WHO Regional Databases were searched to identify experimental and observational studies on the topic. Papers were also identified by hand searching references from included studies. No language or date restrictions were applied in the search. The findings were presented at international meeting [18,19] and shared with professionals in the relevant fields of maternal and child health, following which results were updated based on current searches and expert opinion. Studies were included if they reported the effectiveness of interventions for prevention and management of preconception infections on MNCH outcomes. Methodology is described in detail elsewhere [20].

For the studies that met the final inclusion criteria, we abstracted data describing study identifiers and context, study design and limitations, intervention specifics and outcome effects into a standardized abstraction form. The quality of experimental studies were assessed using Cochrane criteria [21], whereas STROBE guidelines were used to assess the quality of observational studies [22]. We conducted meta-analyses for individual studies and pooled statistics was reported as the odds ratio (OR) and relative risk (RR) between the experimental and control groups with 95% confidence intervals (CI). Mantel–Haenszel pooled RR and corresponding 95% CI were reported or the Der Simonian–Laird pooled

RR and corresponding 95% CI where there was an unexplained heterogeneity. All analyses were conducted using the software Review Manager 5.1 [23]. Heterogeneity was quantified by Chi^2^ and I^2^, in situations of high heterogeneity, causes were explored and random effect models were used.

## Results

**Figure 1:**
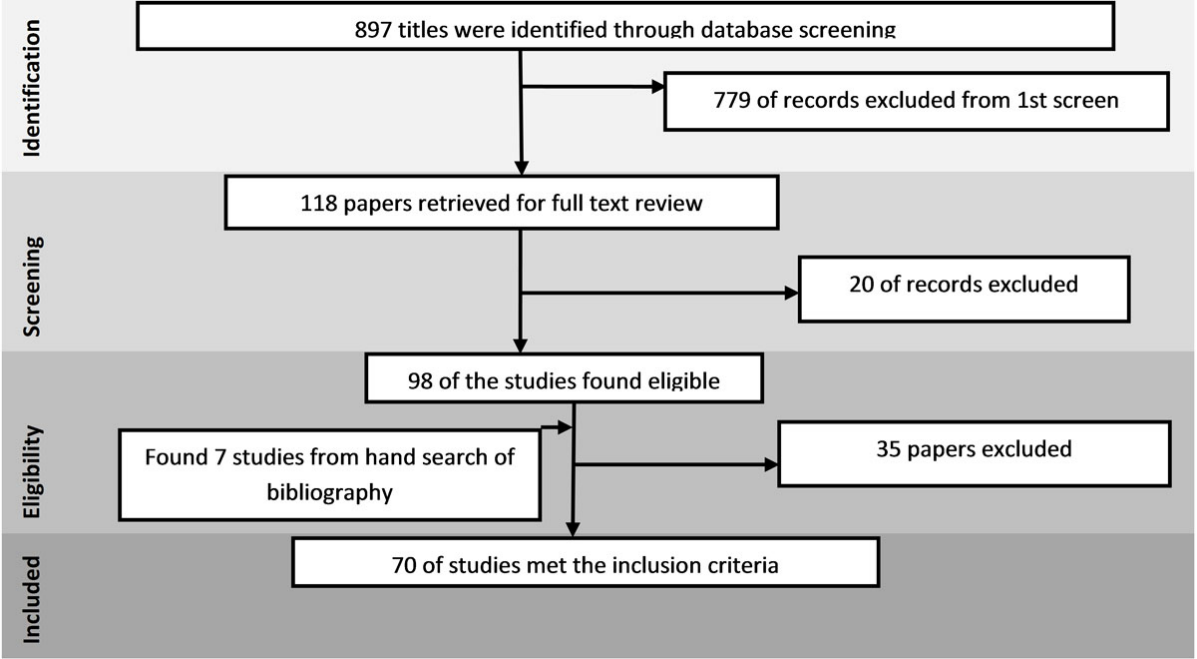
Search flow diagram

The review identified 897 papers from search in all databases. After the initial title and abstract screening, 118 full texts were reviewed to identify papers which met the inclusion criteria and had the outcomes of our interest. Seventy studies were finally selected for abstraction and analysis (Figure 1). Information related to each included study can be found on the following link: https://globalmotherchildresearch.tghn.org/site_media/media/articles/Preconception_Report.pdf

## Sexually transmitted infections

This review assessed the literature pertaining to the effects of gynecologic infections in women in the preconception period on maternal, newborn and child health (MNCH) outcomes and interventions intended to reduce these infections and hence any associated morbidity/mortality. One essential point to keep in mind is the great overlap between interventions targeting STIs, HIV, teenage pregnancies and unwanted pregnancies (The latter two are discussed in detail elsewhere [24]). As far as was possible the data found was disaggregated to focus only on the effect of STIs.

**Figure 2:**
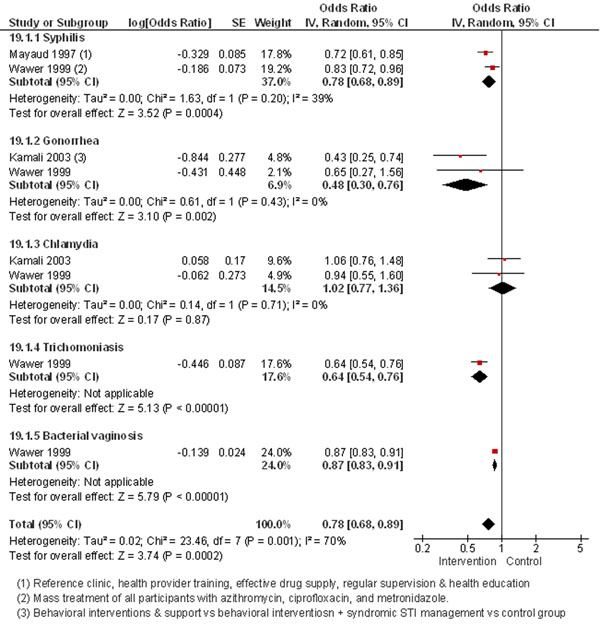
**Prevalence of STDs in mass treatment vs. placebo: evidence from controlled trials** Citations to the included studies: Mayaud 1997 [26], Wawer 1999 [27], Kamali 2003 [25]

The review identified 10 intervention studies [25-34]. The pooled analysis of three interventions studies [25-27] showed that post-intervention STI prevalence significantly decreased by 22% (Figure 2). Behavioral treatments in conjunction with STI management reduced the incidence of gonorrhea by 57% [25]. Healthcare interventions increasing access and availability of STI management led to a significant decrease in syphilis [26]. Mass treatment with antibiotics significantly dropped the rates of syphilis, trichomoniasis and bacterial vaginosis [27].

For behavioral interventions, re-infection or new STI rates significantly declined (OR 0.65 95% CI 0.53-0.80) at 1 year after the intervention [32-34]. The Magnolia Case Management project also showed significant reductions in the incidence and prevalence of STIs by educating women about well-woman care and making healthcare more accessible [28]. Schillinger et al. [29] found a non-significant 20% decrease in the risk of re-infection, with Chlamydia, among women in the patient delivered partner treatment arm than among those in the self-referral arm. On the other hand, Branson et al. [30] did not report any difference in the rate of new STIs among those receiving information, motivation and skills versus those receiving standard counseling. Similarly Boyer et al. [31] found no difference in new STIs six-months post skills sessions versus standard risk-reduction counseling.

**Figure 3:**
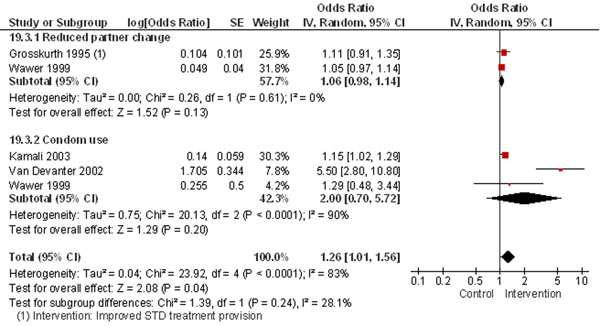
**Safer sexual behavior in Intervention vs. Control groups: evidence from controlled trials** Citations to the included studies: Grosskurth 1995 [35], Wawer 1999 [27], Kamali 2003 [25], Van Devanter 2002 [36]

Most studies reviewed for interventions for STI control reported outcomes related to safer sexual behaviors. The analysis showed interventions promoted overall safer practices in the subjects especially in terms of a two-fold increase in condom use (Figure 3) [25,27,35,36]. Other studies also showed improved condom use after motivational, skill-based interventions [30,31]. In Thailand STI rates have been successfully reduced through enforced condom use [37].

## HIV/AIDS prevention strategies

Although half of women who are pregnant and HIV-positive receive ART, little data exists on prevention of mother-to-child transmission (PMTCT) through interventions before pregnancy [38].

Reducing the burden of HIV in women of reproductive age will prevent transmission of the virus to the next generation and ensure that children do not lose their mothers to AIDS. This review assessed studies of any intervention in women age 15-45 who were not currently pregnant, that improved MNCH outcomes or reduced the incidence of HIV. It was previously postulated that participants in HIV prevention efforts might perceive their risk for transmission to be reduced. Multiple studies have since confirmed that various interventions- including risk reduction [39], ART [40], post-exposure prophylaxis [41], and voluntary counseling and testing (VCT) [42] increase safe sexual practices, even in people who are HIV-positive [43] which would presumably reduce HIV transmission. Hence this review also includes HIV preventive interventions that showed an impact on safe sex behaviors. Since the outcome of interest was reduced transmission, the review also included studies in which the outcome was incidence in men of reproductive age. The review did not, however, include studies where couples used assisted reproductive technologies (such as intracytoplasmic sperm injection or sperm washing) to conceive, since such procedures are expensive and not yet accessible to the population in general, even though they minimize the risk of transmission. Although the risk of transmission is much higher in certain groups such as commercial sex workers and intravenous drug users, the review only described some studies. Thus public health programs to prevent HIV must target men and women, adolescents and adults, couples and individuals, as well as focus more intensive efforts at high-risk populations and their partners.

**Figure 4:**
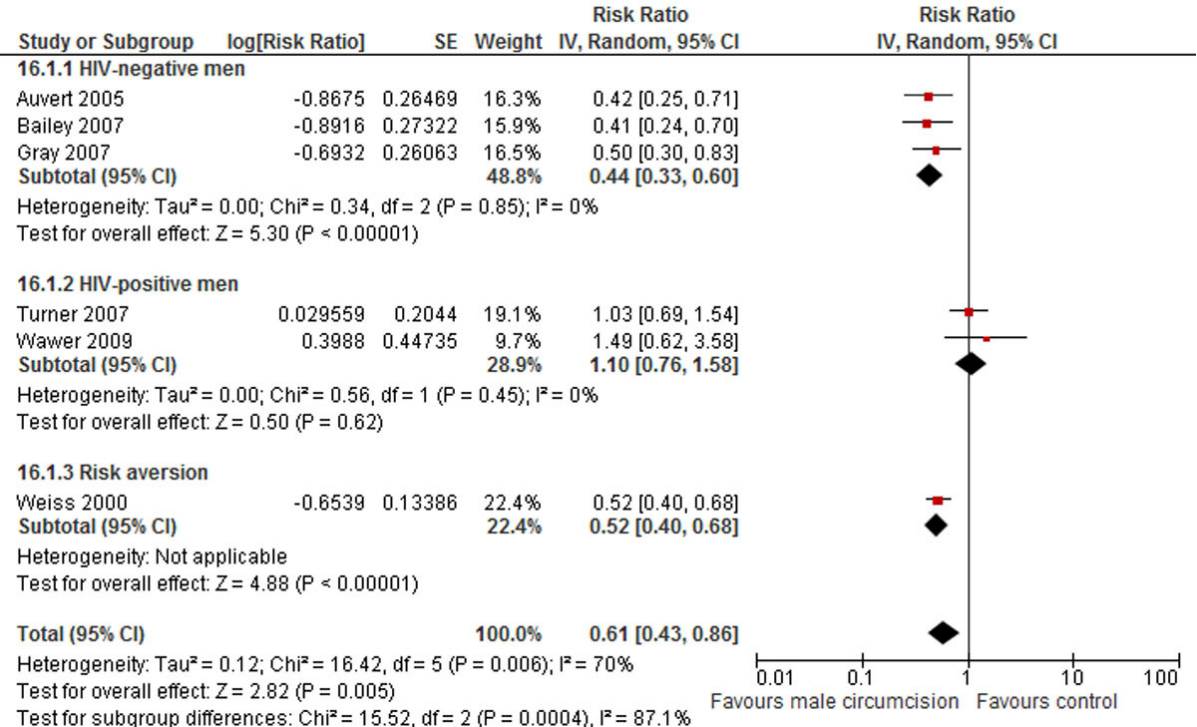
**Male circumcision and the risk of HIV infection: evidence from controlled trials** Citations to the included studies: Auvert 2005 [47], Bailey 2007 [48], Gray 2007 [49], Wawer 2009 [50], Weiss 2000 [51]

The review identified 55 trials [25,27,32,35,44-94]. Studies that reviewed the impact of pre-exposure prophylaxis (PrEP) [45,46], which entails the seronegative partner using antiretroviral drugs, especially tenofovir around the time of conception to minimize the risk of transmission, found an incidence rate ratio of 0.35 (95% CI: 0.03-1.93) for HIV/AIDS, however, the trial lacked study power due to inadequate person-years of follow-up. On the other hand, ART for people who are HIV-positive (treatment as prevention) has consistently been shown to lower the incidence rates of HIV, not just among serodiscordant couples [44], but even in the entire population. As a rather proximal intervention, this review found that male circumcision significantly reduces the risk of acquiring HIV (RR 0.49; 95% CI 0.40-0.59), but is not effective in preventing transmission from HIV-positive men to their partners (RR 1.10; 95% CI: 0.76-1.58) (Figure 4) [47-51].

**Figure 5:**
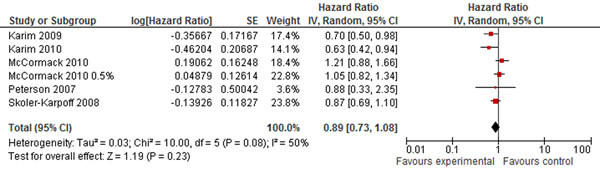
**Microbicides and the risk of HIV infection: evidence from controlled trials** Citations to the included studies: Karim 2009 [52], Karim 2010 [53], McCormack 2010 [54], Skoler-karpoff 2008 [55], Peterson 2007 [46]

**Figure 6:**
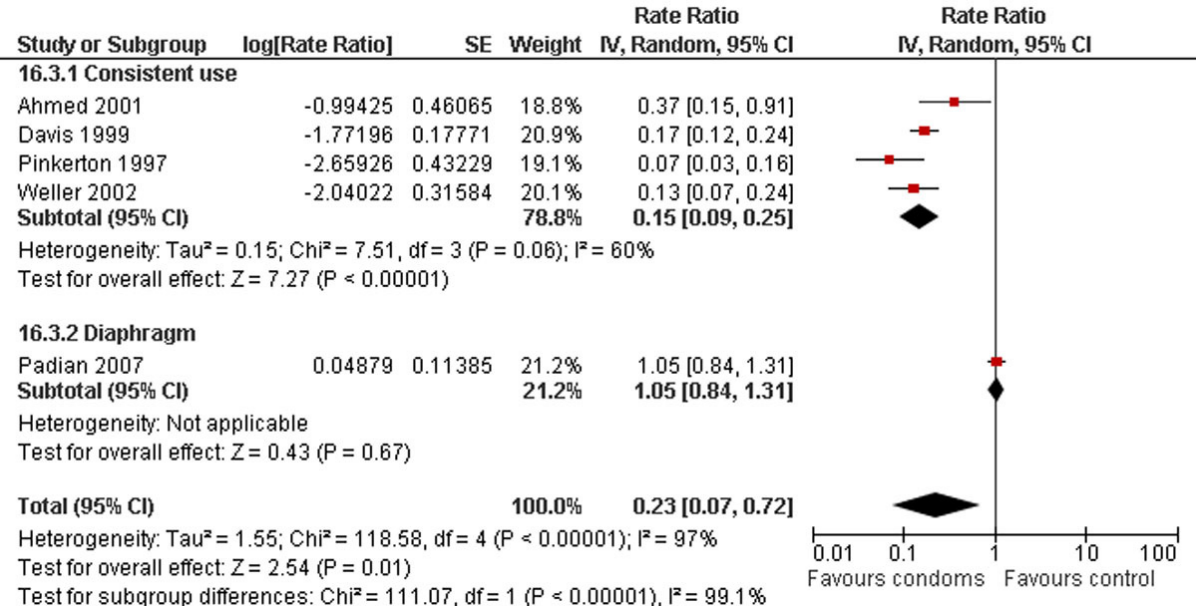
**Barrier methods (especially condom use) and the risk of HIV: evidence from controlled trials** Citations to the included studies: Ahmed 2001 [56], Davis 1999 [57], Pinkerton 1997 [58], Weller 2002 [59], Padian 2007 [60].

Studies also showed that microbicides non-significantly increases the risk of HIV infections (HR 0.89; 95% CI: 0.73-1.08) (Figure 5) [46,52-55]. Whereas, condom use during intercourses decreases the risk by 77% (RR 0.23; 95% CI: 0.07-0.72): (Figure 6) [56-60].

**Figure 7:**
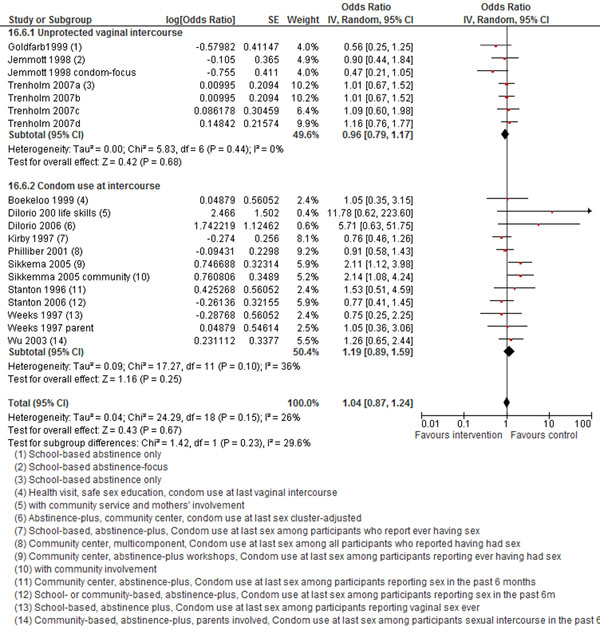
**Interventions to prevent HIV in adolescents: evidence from controlled trials** Citations to the included studies: Goldfarb 1999[65], Jemmott 1998[66], Trenholm 2007[67], Boekeloo 1999[68], Dilorio 2006[69], Kirby 1997[70], Philliber 2001[71], Sikkema 2005[72], Stanton 1996[73], Stanton 2006[74], weeks 1997[75], Wu 2003[76].

The review also pooled intervention trials that reported whether intercourse (especially vaginal) was protected through use of condoms. Meta-analysis showed that interventions did not significantly affect adolescents’ condom use during intercourse (OR 1.04; 95% CI 0.87-1.24) [65-76], however, these results must be interpreted cautiously since the outcome was not uniformly defined (Figure 7).

**Figure 8:**
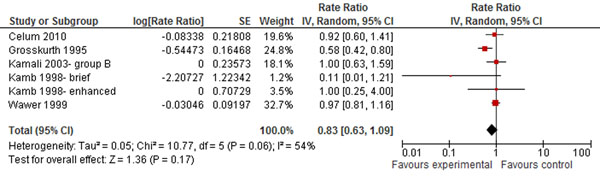
**Management of STIs and the risk of HIV: evidence from controlled trials** Citations to the included studies: Celum 2010[64], Grosskurth 1995[35], Kamali 2003[25], Kamb 1998[32], Wawer 1999[27].

Strong empirical evidence illustrates that other STIs, especially ulcerative diseases and HSV-2, promote HIV transmission with risk increased by 2-5 times that in the general population [95,96]. STIs can therefore interfere with the effectiveness of other interventions to prevent HIV [97]. Management of STIs, including screening, counseling and treatment, has been shown to reduce the risk of HIV. This review found a non-significant slightly decreased risk (RR 0.83; 95% CI 0.63-1.09) since it only included arms of factorial trials in which STI management was the only difference from the other trial arms (Figure 8) [25,27,32,35,64].

Behavioral interventions to reduce the risk of HIV are heterogeneous and target different populations [87,89], but broadly may incorporate HIV/AIDS education, condom promotion and skills, peer educators, skills to negotiate safe sexual behavior, address sociocultural barriers and personal risk reduction, counseling and testing. Overall, these interventions showed a beneficial impact through reduction of risky sexual behaviours, and on decreased STI incidence [86]. It appeared that interventions are more effective for HIV-positive individuals [84,85,98] and serodiscordant couples as well as high-risk ethnic populations [77-79,81,82,90]; and if they are multicomponent, based on cognitive-behavioral theory and provide participants with the skills to ensure safe sexual practices. It remains unclear whether interventions have more effect if targeted specifically by gender. Amongst intravenous drug users, interventions (except counseling) to prevent HIV infection do result in reduced injection and sexual risk behavior [80,88,91,92,94]. Risk reduction in this high-risk population through harm reduction, substitution treatment, and peer education is important to prevent transmission to the rest of the population.

## Vaccine usage pre-conceptionally

Although vaccination has been a highly successful public health campaign, gaps remain in coverage. Immunization during the preconception period can prevent many diseases which may have serious consequences or even prove fatal to the mother or newborn. For example, rubella exposure during early pregnancy can result in pregnancy loss, stillbirths or congenital rubella syndrome. Further, live-virus vaccines are recommended in the preconception period because they cannot be safely administered during pregnancy; others have maternal benefits because they avoid treatment that might have adverse consequences for the pregnancy.

The review intended to look at the feasibility of vaccination of women while they are contemplating a pregnancy, focusing on how this may further decrease the morbidity and mortality associated with gestational infections and how such vaccination could be successfully implemented.

**Figure 9:**
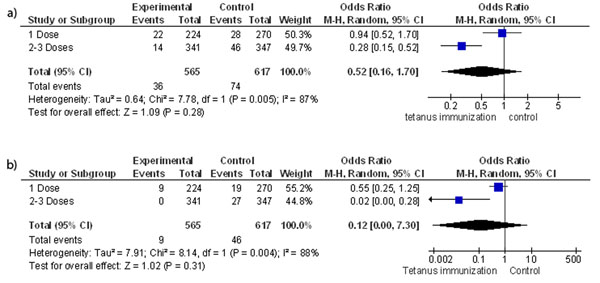
**(a) Women immunized with Tetanus toxoid versus influenza vaccine and odds of neonatal deaths (b) Women immunized with Tetanus toxoid versus influenza vaccine and odds of tetanus specific neonatal deaths** Citation to included study: Newell 1966[101]

Four intervention trials were found that assessed the effectiveness of tetanus toxoid vaccination in women of child-bearing age [99-115]. Analysis showed that vaccination against tetanus averted a significant number of neonatal deaths (including those specifically due to tetanus) when compared to placebo in women receiving more than 1 dose of the vaccine (OR 0.28; 95% CI: 0.15-0.52); (OR 0.02; 95% CI: 0.00-0.28) (Figure 9) respectively [101]. This was also true for tetanus-diphtheria toxoid (OR 0.52; 95% CI: 0.29-0.91) [99]. These findings were confirmed by observational data from mass immunization programs of several countries [100,102,116] and a review [117]. However, no trials were located that compared preconception vaccination with immunization done during pregnancy.

The evidence for preconception vaccination against rubella was from separate interventional studies for screening and vaccination, and from observational data of national vaccination campaigns. Antibody screening is not advised before vaccination since it has a high rate of false negatives [104,107]. Premarital screening increases the rates of vaccination only when providers advise vaccination and offer it directly after counseling, or other motivation is provided with screening, such as a letter or brochure [106,110,111,113]. National vaccination campaigns for girls and women are cost-effective or cost-saving, and even if vaccination occurs within a few months before preconception, the risk of the fetus developing congenital rubella syndrome from vaccination is at most 1.7% [103,105,108,109,112,114,115]. In only one trial was the rate of neonatal death higher in the vaccination arm (1.2% versus 0% in controls). Finally, if women are found to be non-immune after delivery, it is advisable that they be vaccinated in the postpartum period to provide protection for the subsequent pregnancy.

The advantage of administering the HPV vaccine to prevent cervical cancer means that girls must be vaccinated before the onset of sexual activity [118]. HPV vaccination provides further advantage, however, to young women and their newborns by reducing the possibility of preterm birth due to cervical incompetence and the rate of laryngeal papillomatosis in the newborn [119]. In phase 3 clinical trial and post-licensure surveillance, the only significant difference in neonatal outcomes was found for miscarriage when Cervarix was administered within 3 months preconception [120,121].

## Periodontal disease and dental caries

Preterm birth and LBW is a leading cause of neonatal and infant mortality and morbidity. In attempting to reduce this burden of disease, it was first necessary to understand the mechanism by which preterm birth occurs; Goldenberg et al. [122] was the first to suggest that infection of the maternal-fetal membranes was responsible for early spontaneous preterm birth. While it was easily conceivable that direct infection, for example from bacterial vaginosis, could lead to preterm labor, around the same time Offenbacher et al. [123] demonstrated that periodontitis was also a risk factor for preterm birth.

Given the surprisingly high odds (OR 7.5) that maternal periodontal disease could result in preterm LBW babies, researchers sought to confirm this effect and examine whether improving maternal oral health would improve pregnancy outcomes. Systematic reviews incorporating epidemiologic and interventional evidence have not consistently supported the association [124-127]. Meta-analyses of risk aversion, however, seem to acknowledge the relationship (OR for association with preterm LBW ranging from 2.83-4.28; and OR for association with other adverse pregnancy outcomes including miscarriage, intrauterine growth restriction, gestational diabetes and preeclampsia range from 1.10-20.0) with reservation [128-130]. Further, clinical trials that assess periodontal treatment have found differential effects on pregnancy outcomes [131]. Reviews [132,133] seeking to explain these inconsistencies have cited lack of uniform definitions for exposure and outcomes; failure to control for confounders that are known risk factors for preterm birth; the use of just a single session of treatment; and the possibility that ameliorating this risk might only improve outcomes in a subpopulation.

Since periodontal infection is presumably chronic, it is reasonable to suppose that prevention and/or treatment before pregnancy might help women maintain good oral health during pregnancy and prevent adverse outcomes. However, most risk-aversion studies and clinical trials have been conducted during pregnancy.

Oittinen et al. 2005 [134] was the only study found that exclusively focused on pre-pregnancy periodontal infection and adverse pregnancy outcome (miscarriage and preterm birth not disaggregated) and showed an OR 5.5 (95% CI 1.4-21.2). Interestingly they found no effect for dental caries (OR 1.0). A cohort study [135] was excluded since periodontal treatment or prevention were not explicitly stated as being provided before pregnancy.

## Cytomegalovirus

Cytomegalovirus (CMV) is the most common congenital viral infection, and is a leading cause of congenital deafness and neurodevelopmental disability. Between 0.2 and 2.5% of all live newborns are infected [136]. Ten percent of these will be symptomatic at birth, and of the remaining, another 10% will also go on to develop disease sequel [137]. Managing children with the permanent consequences of congenital CMV costs over $300,000 annually per child, and more children suffer from such outcomes each year than from any other congenital defect [136]. The annual seroconversion rate for pregnant women is 2.3% [138], and the greatest risk is incurred by newborns whose mothers acquire the primary infection during pregnancy (1% of pregnancies) since the intrauterine transmission rate is 40% or higher and decreases with increasing gestational age [139].

The propensity for newborns to acquire the infection from their mothers and the devastating consequences of congenital CMV infection has motivated researchers to try and develop a vaccine. Such efforts have been hampered, however, by confusion as to whether maternal immunity actually provides protection for the fetus- 60% of infants with CMV are born to mothers who were immune before pregnancy [140]. This review, therefore, examined the neonatal outcomes for women who were infected pre-pregnancy, and therefore developed immunity to CMV.

The studies pertaining to preconceptional immunity to CMV and fetal infection were all observational (including cohort) studies. In one study [141], of 46 newborns to women with preconceptional immunity, 16 were infected with CMV. Sixty two percent (62%) of the mothers with infected infants versus 13% of those with uninfected infants had acquired new antibody specificities, indicating that maternal reinfection with a different strain of CMV could still lead to congenital infection.

Fowler et al. [142] showed that preconceptional immunity (seropositive at a previous birth) resulted in a significantly lowered risk (RR 0.31) of infection in the newborn. Shaamash et al. [143] also showed that preconceptional (not clearly defined, since blood sample taken during antenatal visit) immunity ameliorates disease, even if it does not block transmission with infants of 132 seropositive women all being asymptomatic- this includes 2 infant-mother pairs with recent infection.

Further research tried to delineate whether the timing of the primary infection in relation to conception was a risk factor: Daiminger et al. 2005 [144] showed that women with primary infection 2 months to 2 weeks pre-pregnancy did not have infected infants, whereas women with primary infection in 1 week before to 1 month after conception had similar rates of transmission as those women acquiring CMV during pregnancy. These results are somewhat misleading, however, since exposure for 10 women could not be definitively categorized as preconceptional or periconceptional. The distinction between primary preconceptional (3 months before) and periconceptional (1 month after) exposure was also made by Revello & Gerna 2002 [145] with a higher rate of congenital infection in the periconceptional exposure group. Fowler et al. 2004 [146] also demonstrated that among mothers who seroconverted between pregnancies, the risk was greatest for those with birth intervals <24 months. Moreover, the risk may also depend on endemicity, indicated by maternal sero-prevalence rates [147].

More recently, Revello et al. 2006 [148] showed that of 14 women who had primary CMV infection 2 weeks to 4.5 months before pregnancy, only 1 had an infected newborn (another 1 terminated her pregnancy). Hadar et al. 2010 [149] confirmed these results (periconception defined as 1 month prior to 3 weeks after conception) in a larger group of women with primary infection. Zalel et al. 2008 [150] however, studied 6 women with preconceptional immunity, all of whom had severely infected fetuses, proving that recurrent infection can be as hazardous as primary infection in pregnancy.

## Discussion

It is well known that the best time to identify and address risk factors for poor reproductive health outcomes for mothers and babies is not after but before conception through preconception care [98]. Infections are one of those risks, because certain infectious diseases carry a real threat to mothers and the foetus in utero. STI are serious global reproductive health problem, the burden is high among women from poor socio-economic status. This review identified that mass treatment of STIs with antibiotics leads to a 22% reduction in its prevalence, and behavioral/counseling interventions, on the other hand, led to a 35% decrease in STI incidence. Interventions targeting STIs led to a significant 26% increase in condom use. This finding is in line with the systematic review [151] on effectiveness of condoms in reducing STIs like chlamydia and gonorrhea. A Systematic review by Shepherd et al. [152] on the effectiveness of behavioral interventions for prevention of STIs in adolescents and young adults also showed that behavioral programs bring about increase in knowledge and self-efficacy and changes in behavioral outcomes to a lesser degree. The review did not study the effects on MNCH outcomes, henceforth, it concluded that such school-based skills and information interventions play a significant role in improving overall knowledge about the subject, foster favorable attitudes and ‘behavioral intentions’.

Studies that assessed the impact of PrEP found non-significant lower incidence of HIV/AIDS, similarly, ART also found lower incidence of HIV/AIDS. However, concerns with PrEP include adherence, the risk of developing resistant viral strains, safety, cost and behavioral risk compensation. Condom use is scientifically proven to radically diminish the risk of HIV transmission, and condoms have the additional advantage of protecting against other STIs and unintended pregnancy [153]. Since other contraceptive methods, especially those that are female-dependent, are not effective, there is a real need for methods to increase condom use among serodiscordant partners and other individuals that are high-risk for HIV transmission [154]. Research should now focus on developing effective interventions, assessed through rigorous methods, to promote the use of condoms during all sexual exposures. It was also hoped that microbicides might provide a way for women to control their risk for HIV infection, however, microbicides do not provide protection from HIV and might even increase harm through increased risk of genital ulceration and injury. Voluntary counseling and testing, on the other hand, has also not shown to reduce the risk of transmission through unprotected intercourse; however it is still advocated for individuals to determine their serostatus, in order to better protect themselves and others [61-63]. Behavioral interventions showed a beneficial impact through reduction of risky sexual behaviors, and on decreased STI incidence. However, the reduction in HIV incidence was less convincing. The lack of consistent effect across studies might be due to differing sites and populations [83], and the use of different control groups. .

While there is a definite need for more HIV prevention interventions that are specifically effective in women, reducing HIV incidence in the general population will decrease the probability that women are exposed to HIV. Ongoing trials may provide evidence for pre-exposure prophylaxis and prove that treatment is effective as prevention. Men who are circumcised halve their chances of becoming infected, and of further transmitting it to their female partners STI, especially ulcerative types such as HSV-2, significantly increase the risk of becoming HIV-infected. However, pooling results of participants in randomized trials who only differed from the controls in terms of STI management did not yield significant evidence of effect. Screening and management of STIs is still promoted, because individuals with STIs have both increased biologic and behavioral risk. The components of behavioral interventions that increase likelihood of success in preventing HIV have been documented; however non-uniform reporting of outcomes limits comparison of effect for populations and high-risk groups. Even in endemic regions, such as sub-Saharan Africa, there have been few interventions carried out in youth, who are at high-risk of HIV infection [155]. Proof of efficacy must now be translated into effectiveness, through replication of successful interventions in various contextual settings and target populations, and reporting of standardized and biologic outcomes (especially HIV incidence).

A number of best practice interventions have been identified to prevent HIV infection in high-risk individuals [93]. Adolescents are a special group with unique social influences, and are at extremely high risk. Many reviews have been conducted in this area, but data synthesis has tended to be qualitative, or has focused on a single type of intervention. It is crucial to note here that interventions which aim to prevent STIs including HIV, and teenage and unintended pregnancies, overlap to a large extent. Further, there is a lack of uniformity in the outcomes that trials report- for instance, some report STI incidence and others prevalence or repeat infections; and some discuss unprotected intercourse while others assess condom use at last intercourse. On the other hand, such outcomes may have been assessed in more than one way to ensure response accuracy. Surprisingly few trials report public campaigns as an intervention or HIV incidence as an outcome, despite evidence to show the high rates of infection and risky sexual behavior among teens. Preconception counseling should be offered to women of reproductive age as soon as they test HIV-positive, and conversely women of reproductive age should be screened with their partners before pregnancy. While many interventions have been tested they mostly look at endpoints such as safer sexual behavior. These would eventually have an indirect effect on possibly reducing adverse pregnancy outcomes, however the need of the hour are epidemiologic studies that better address the issue at hand - reducing STIs in women in the preconception period to have immediate and large impacts.

Tetanus vaccination (with Tdap) of women of childbearing age has been found to be effective in reducing neonatal deaths from the disease (48%), especially when immunization is complete. Immunization during pregnancy with tetanus toxoid is the general practice in current obstetrics guidelines. All women of reproductive age should receive immunization against rubella if they have no evidence of immunity. Rubella vaccination before pregnancy is safe, even in the periconception period, and protects newborns from congenital rubella syndrome. Clinical trials of HPV vaccination in the preconception period have been shown to be safe, and as national campaigns immunize more women, further evidence of benefit on preterm birth might be found. The effects of pre-pregnancy immunization on MNCH outcomes need to be compared with immunization during pregnancy. Also the duration for which these may be efficacious should be investigated, so that if necessary, women receive booster vaccinations before subsequent pregnancies.

While it is tempting to extrapolate the evidence for periodontal treatment during pregnancy to the preconception period, high level evidence is still lacking to prove that prevention or treatment of periodontal disease before or during pregnancy consistently prevents adverse outcomes. Further large-scale randomized controlled trials are necessary to establish that such therapy is warranted. Further, it must be noted that like many other interventions, such therapy might need to be a process that is instituted before, but continues throughout pregnancy, in order to achieve the maximum benefit. Currently, preconception screening and treatment of periodontal disease can only be recommended to improve women’s oral health.

Although the evidence is still far from concrete, it appears that preconceptional immunity does provide some protection to the fetus from CMV infection. However, recurrent or periconceptional maternal infection are as risky as infection during pregnancy. For the same reason, and due to cost constraints, maternal screening is also not advised unless the woman is symptomatic or there is evidence of fetal infection. While observational studies with larger sample sizes may provide clarity as to whether a vaccine could be effective, women of reproductive age should be counseled on how to reduce their exposure to CMV around pregnancy [136,139]. Young children are the main source of CMV infection, and therefore women planning to conceive should be counseled to avoid contact with children’s saliva or urine, and wash hands thoroughly if such contact occurs. Further, women of reproductive age diagnosed with primary CMV infection should be counseled to delay pregnancy, although the minimum interval is not yet clear [137]. Some evidence also suggests that CMV hyper immune globulin could be administered for both therapeutic and preventive purposes [156].

## Conclusion

It is very important to address these infectious diseases in preconception period. Risk assessment, screening, and treatment for specific infections should be a component of preconception care because there is convincing evidence that treatment of these infections before pregnancy prevents neonatal infections; consequences to the developing fetus (syphilis); or transmission of an infectious agent with potential for chronic infection of the offspring (HIV). Given the association of periodontal disease with preterm birth in observational studies, trials to evaluate specifically the effect of preconception treatment interventions for these conditions are warranted.

## Competing interests

We do not have any financial or non-financial competing interests for this review.

## Peer review

Peer review reports are included in additional file 1.

## Supplementary Material

Additional file 1 Peer review reports. Click here for file

## Declarations

This article has been published as part of *Reproductive Health* Volume 11 Supplement 2, 2014: Preconception interventions. The full contents of the supplement are available online at http://www.reproductive-health-journal.com/supplements/11/S3. Publication charges for this collection were funded by the Partnership for Maternal, Newborn & Child Health (PMNCH).
