# Supplementary material for: Preconception care: preventing and treating infections
Source: Reprod Health. 2014 Sep 26;11(Suppl 3):S4. doi: 10.1186/1742-4755-11-S3-S4 (PMC4196562; doi:10.1186/1742-4755-11-S3-S4)
Supplement: Additional file 1 — Peer review reports. [file 1742-4755-11-S3-S4-S1.pdf]

Referee's comments to the authors– this sheet WILL be seen by the author(s) and published with the article

Title	**Preconception - Preventing and treating infections**
Author(s)	Zohra S Lassi, Ayesha M Imam, Sohni V Dean, Zulfiqar A Bhutta
Referee's name	Farah Qamar

When assessing the work, please consider the following points, where applicable:

1. **Is the question posed by the authors new and well defined?**
2. **Are the methods appropriate and well described, and are sufficient details provided to replicate the work?**
3. **Are the data sound and well controlled?**
4. **Does the manuscript adhere to the relevant standards for reporting and data deposition?**
5. **Are the discussion and conclusions well balanced and adequately supported by the data?**
6. **Do the title and abstract accurately convey what has been found?**
7. **Is the writing acceptable?**

Please make your report as constructive and detailed as possible in your comments so that authors have the opportunity to overcome any serious deficiencies that you find and please also divide your comments into the following categories:

- Major Compulsory Revisions (which the author must respond to before a decision on publication can be reached)
- Minor Essential Revisions (such as missing labels on figures, or the wrong use of a term, which the author can be trusted to correct)
- Discretionary Revisions (which are recommendations for improvement but which the author can choose to ignore)

Where possible please supply references to substantiate your comments.

When referring to the manuscript please provide specific page and paragraph citations where appropriate.

General comments:

Major compulsory revisions:

Minor essential revisions:

None needed

Discretionary revisions:

(continue on the next sheet)

Referee's comments to the authors– this sheet WILL be seen by the author(s) and published with the article

Title	**Preconception - Preventing and treating infections**
Author(s)	Zohra S Lassi, Ayesha M Imam, Sohni V Dean, Zulfiqar A Bhutta
Referee's name	Sajid Soofi

When assessing the work, please consider the following points, where applicable:

1. Is the question posed by the authors new and well defined?
2. Are the methods appropriate and well described, and are sufficient details provided to replicate the work?
3. Are the data sound and well controlled?
4. Does the manuscript adhere to the relevant standards for reporting and data deposition?
5. Are the discussion and conclusions well balanced and adequately supported by the data?
6. Do the title and abstract accurately convey what has been found?
7. Is the writing acceptable?

Please make your report as constructive and detailed as possible in your comments so that authors have the opportunity to overcome any serious deficiencies that you find and please also divide your comments into the following categories:

- Major Compulsory Revisions (which the author must respond to before a decision on publication can be reached)
- Minor Essential Revisions (such as missing labels on figures, or the wrong use of a term, which the author can be trusted to correct)
- Discretionary Revisions (which are recommendations for improvement but which the author can choose to ignore)

Where possible please supply references to substantiate your comments.

When referring to the manuscript please provide specific page and paragraph citations where appropriate.

General comments:
This is an interesting and important systematic review from a well experienced group in the field. The manuscript is well written and the data adequately presented and discussed. As any other global analysis, the work and results of this paper are, by their nature, broad, but can be informative with more clarity.

Minor essential revisions:

1. The paper needs further clarity whether it reports the issue as a global concern or as a problem of developing or developed countries.
2. The inclusion and exclusion criterion for the data search should be mentioned.
3. The term eligibility and inclusion criteria should be used carefully in Figure 1 on page 3. The flow diagram under the eligibility heading mentions '98 studies found eligible and in the next step its mentioned that 35 studies were excluded as they did not meet the eligibility criteria'. This phenomenon needs to be explained
4. In first line of Methodology section on page 3 the word 'review' is not mentioned after the word 'systematic'.
5. The term 'LBW' should be defined fully in the first line of Periodontal Disease and Dental Caries heading on page 12.

(continue on the next sheet)

Supplement Editor comments

In the Introduction section, there are several assumptions made without giving literature to support such statements. Please provide such references.

Referee's comments to the authors– this sheet WILL be seen by the author(s) and published with the article

Title	**Preconception - Preventing and treating infections**
Author(s)	Zohra S Lassi, Ayesha M Imam, Sohni V Dean, Zulfiqar A Bhutta
Referee's name	Sajid Soofi

When assessing the work, please consider the following points, where applicable:

1. Is the question posed by the authors new and well defined?
2. Are the methods appropriate and well described, and are sufficient details provided to replicate the work?
3. Are the data sound and well controlled?
4. Does the manuscript adhere to the relevant standards for reporting and data deposition?
5. Are the discussion and conclusions well balanced and adequately supported by the data?
6. Do the title and abstract accurately convey what has been found?
7. Is the writing acceptable?

Please make your report as constructive and detailed as possible in your comments so that authors have the opportunity to overcome any serious deficiencies that you find and please also divide your comments into the following categories:

- Major Compulsory Revisions (which the author must respond to before a decision on publication can be reached)
- Minor Essential Revisions (such as missing labels on figures, or the wrong use of a term, which the author can be trusted to correct)
- Discretionary Revisions (which are recommendations for improvement but which the author can choose to ignore)

Where possible please supply references to substantiate your comments.

When referring to the manuscript please provide specific page and paragraph citations where appropriate.

General comments:
This is an interesting and important systematic review from a well experienced group in the field. The manuscript is well written and the data adequately presented and discussed. As any other global analysis, the work and results of this paper are, by their nature, broad, but can be informative with more clarity.

Minor essential revisions:

6. The paper needs further clarity whether it reports the issue as a global concern or as a problem of developing or developed countries.
   The review is a global review and is not limited to any specific region.
7. The inclusion and exclusion criterion for the data search should be mentioned.
   Added in the first paragraph of Methods section.
8. The term eligibility and inclusion criteria should be used carefully in Figure 1 on page 3. The flow diagram under the eligibility heading mentions '98 studies found eligible and in the next step its mentioned that 35 studies were excluded as they did not meet the eligibility criteria'. This phenomenon needs to be explained
   Corrected
9. In first line of Methodology section on page 3 the word 'review' is not mentioned after the word 'systematic'.
   Edited
10. The term 'LBW' should be defined fully in the first line of Periodontal Disease and Dental Caries heading on page 12.
    It is defined on Page 7 under the heading of HIV/AIDS prevention strategies
